# Supplementary material for: Mitochondrial respiration is required to provide amino acids during fermentative proliferation of fission yeast
Source: EMBO Rep. 2020 Sep 7;21(11):e50845. doi: 10.15252/embr.202050845 (PMC7645267; doi:10.15252/embr.202050845)
Supplement: Supplementary file 1 — Expanded View Figures PDF [file EMBR-21-e50845-s001.pdf]

Expanded View Figures

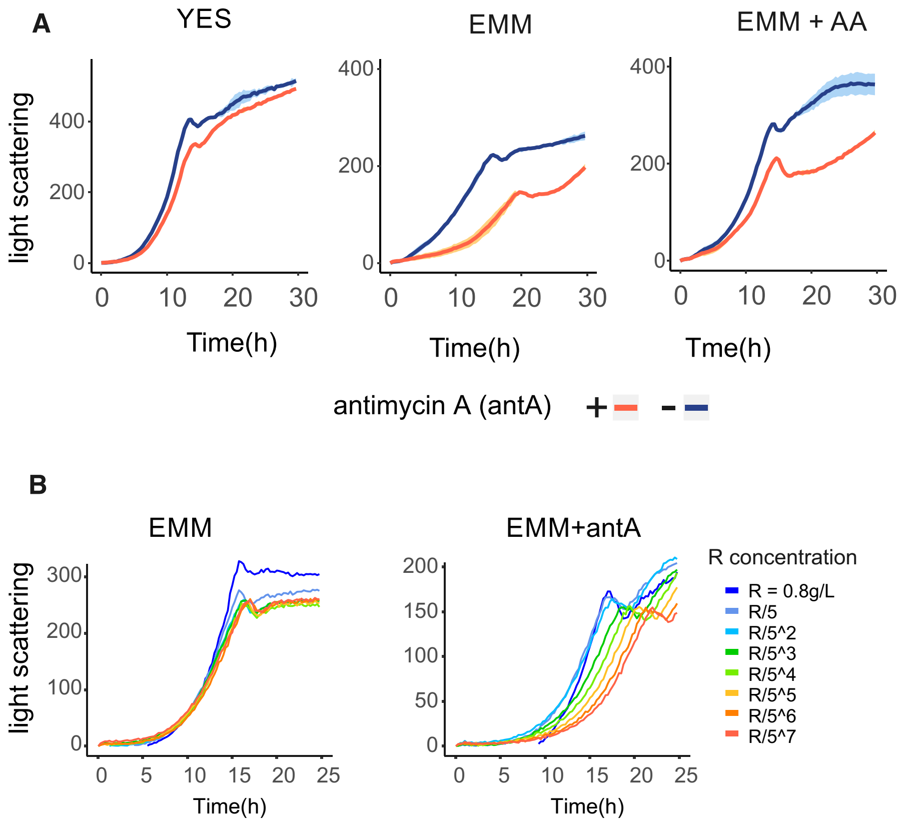

Figure EV1. Arginine improves growth of cells with blocked respiration.

A Growth curves of yeast cultures in rich (YES) or defined (EMM) media, and in defined media supplemented with mix of soluble amino acids (EMM + AA). In each experiment, media were supplemented or not with antimycin A (blue or red curves respectively). Data presented as the mean of three technical repeats (main line) with shades indicating the standard deviation.

B Dose-dependent effect of arginine. Growth curves of yeast cultures in defined media with or without antimycin A (EMM or EMM + antA), supplemented with different amounts of arginine (as indicated in the legend).

 © 2020 The Authors

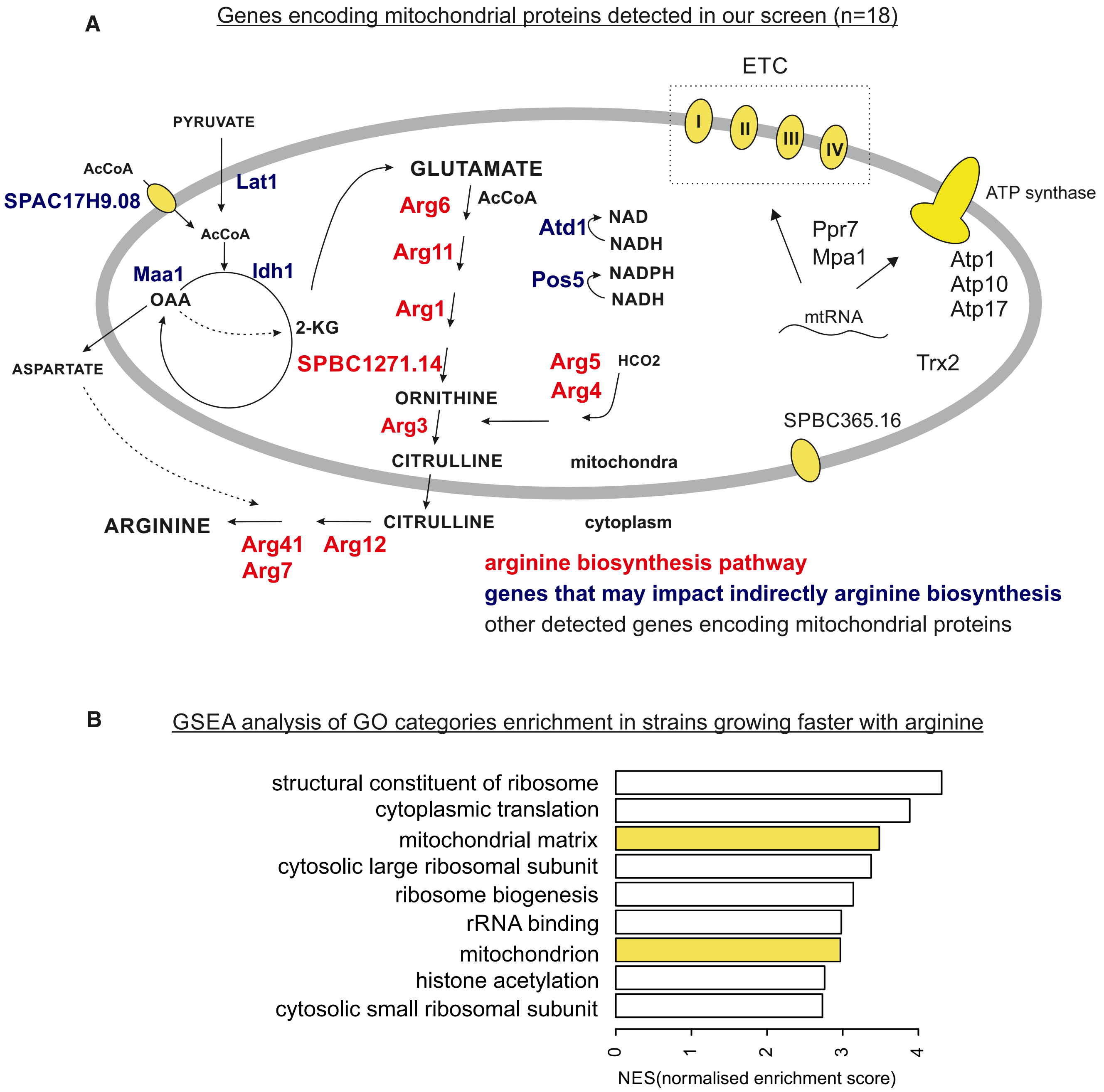

Figure EV2. Genes whose deletion resulted in arginine auxotrophy.

A Genes encoding mitochondrial proteins identified in our screen were separated to ones that are part of arginine biosynthesis pathway (red), ones that may indirectly impact both respiration and arginine biosynthesis (blue) and others (black). Additionally, cytoplasmic enzymes involved in arginine biosynthesis are indicated. Short descriptions of cellular functions as well as budding yeast and human orthologues of genes indicated in the figure are provided in Table EV1.

B Gene Ontology (GO) enrichments in the result of the screen identified using threshold-free GSEA analysis (Subramanian *et al*, 2005). (We show categories enriched among genes whose deletion results in faster growth with arginine; highlighted in yellow are GO categories containing genes encoding mitochondrial proteins).

© 2020 The Authors

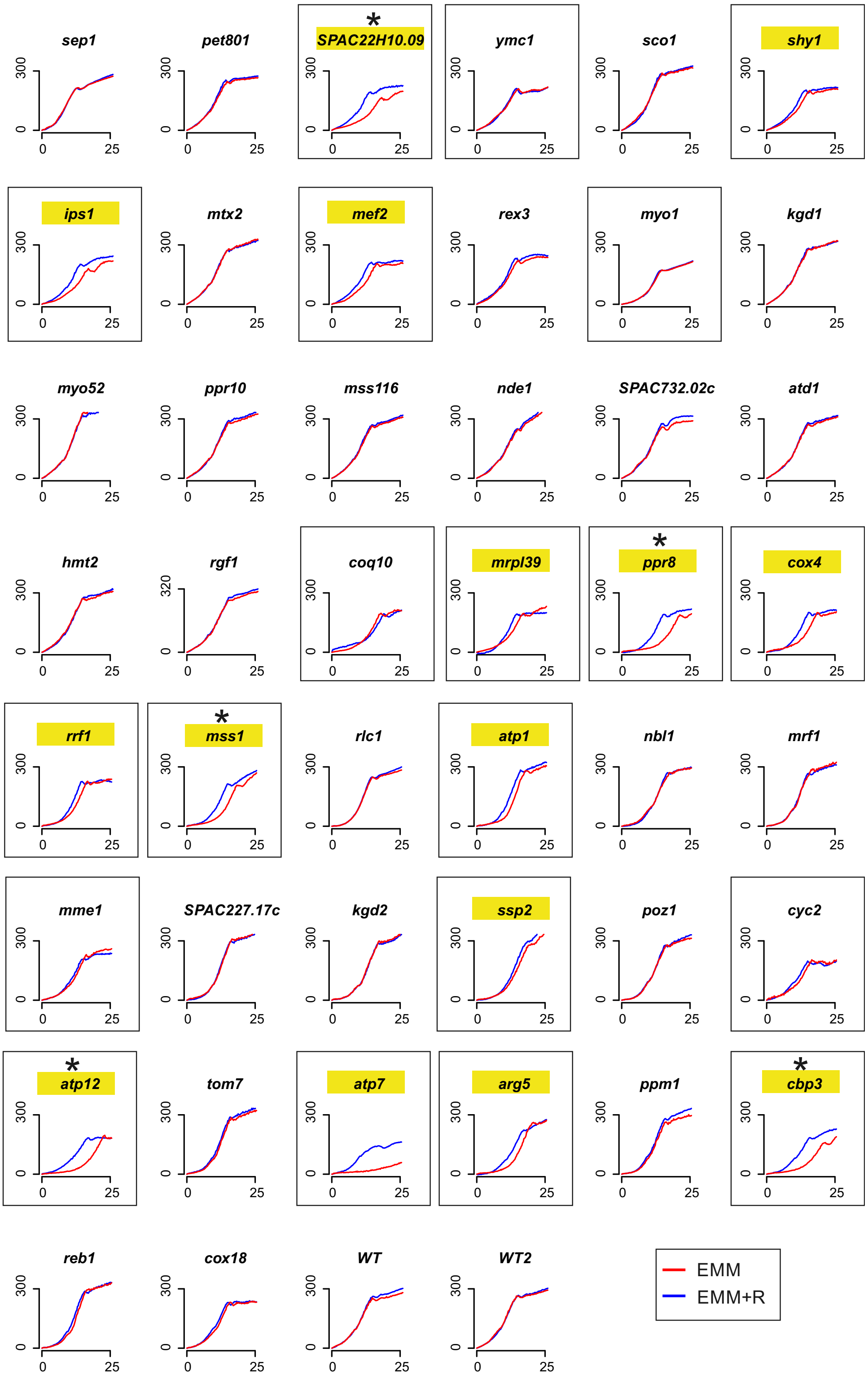

Figure EV3. Growth of respiratory mutants can be improved by arginine.

Growth curves of 44 respiratory mutants in defined media (EMM) with or without arginine (blue or red, respectively). On each plot, the *y*-axis shows the light scattering while the *x*-axis shows the time (hours). Strains were isolated from prototroph library (Malecki & Bähler, 2016). In frames are growth curves of strains whose growth was inhibited in minimal media, and highlighted in yellow are strains whose growth was improved by arginine supplementation. Strains that were selected for further experiments are indicated with asterisks.

 © 2020 The Authors

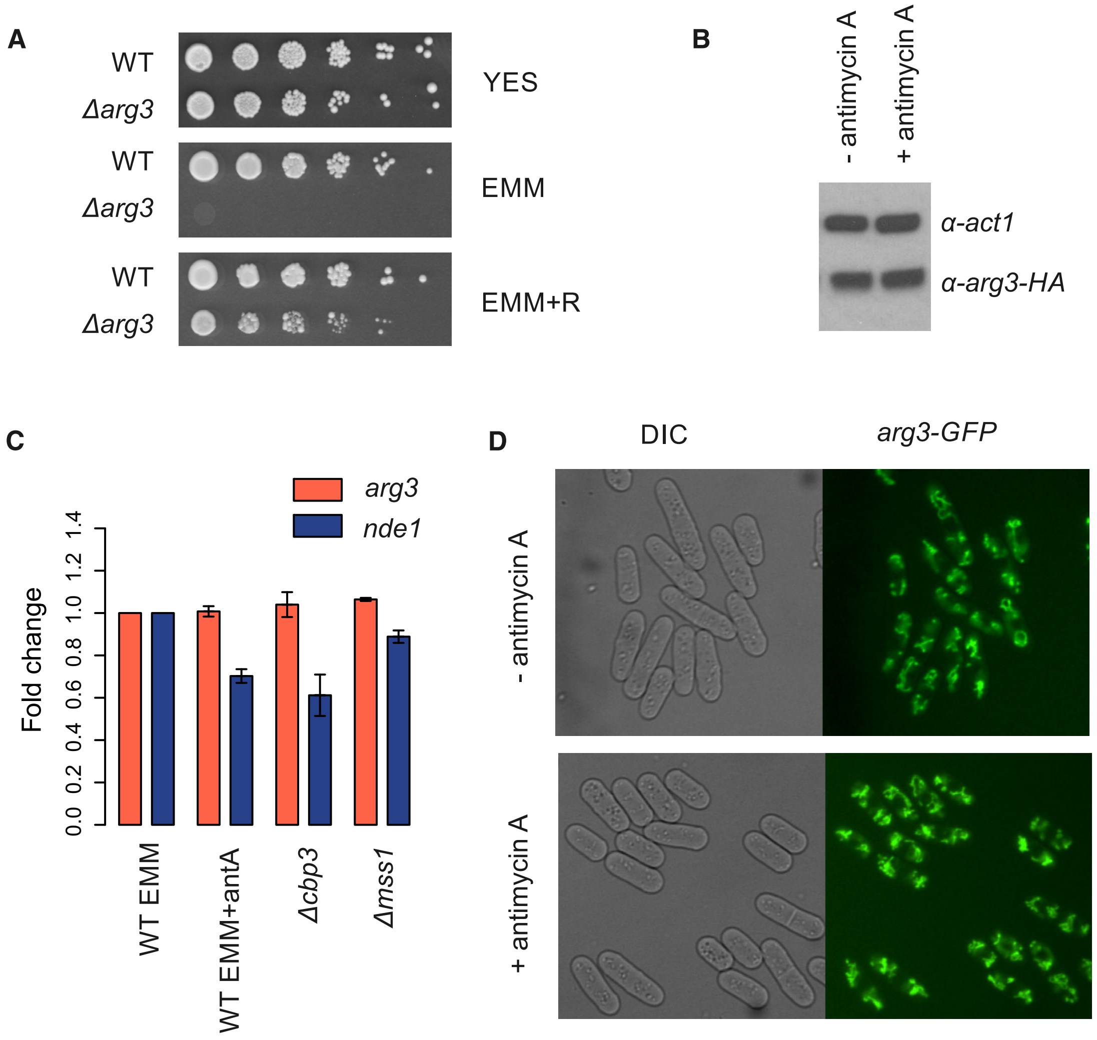

Figure EV4. Respiration inhibition does not impact endogenous *arg3* RNA or protein level.

A Strain with *arg3* gene deletion (*Δarg3*) in prototroph background exhibits arginine auxotrophy. Wild-type prototroph (WT) and *Δarg3* strains were grown in liquid YES media, washed in EMM and spotted on agar plates with rich (YES) and minimal (EMM) medium, and in minimal medium supplemented with arginine (EMM + R).

B Western blot against Arg3-HA fusion protein. Total proteins were extracted from cells grown exponentially in EMM or EMM with antimycin A. Signal obtained using antibodies against actin (α-act1) serves as loading control.

C Level of *arg3* transcript was measured using qPCR method in cells grown in EMM or EMM with antimycin A. Additionally, level of *arg3* transcript was measured in respiratory mutants grown in EMM – *Δcbp1* and *Δmss1*. As a control, we measured level of *nde1* transcript encoding equivalent of complex I electron transport chain that was shown to be repressed in respiratory mutants (Malecki *et al*, 2016). Data were normalised to the *cdc2* transcript level. Data are presented as mean of two independent repeats ± SD.

D Arg3-GFP fusion localises to mitochondria, we detected similar intensity of GFP signal in cells grown in EMM or EMM with antimycin A.

© 2020 The Authors

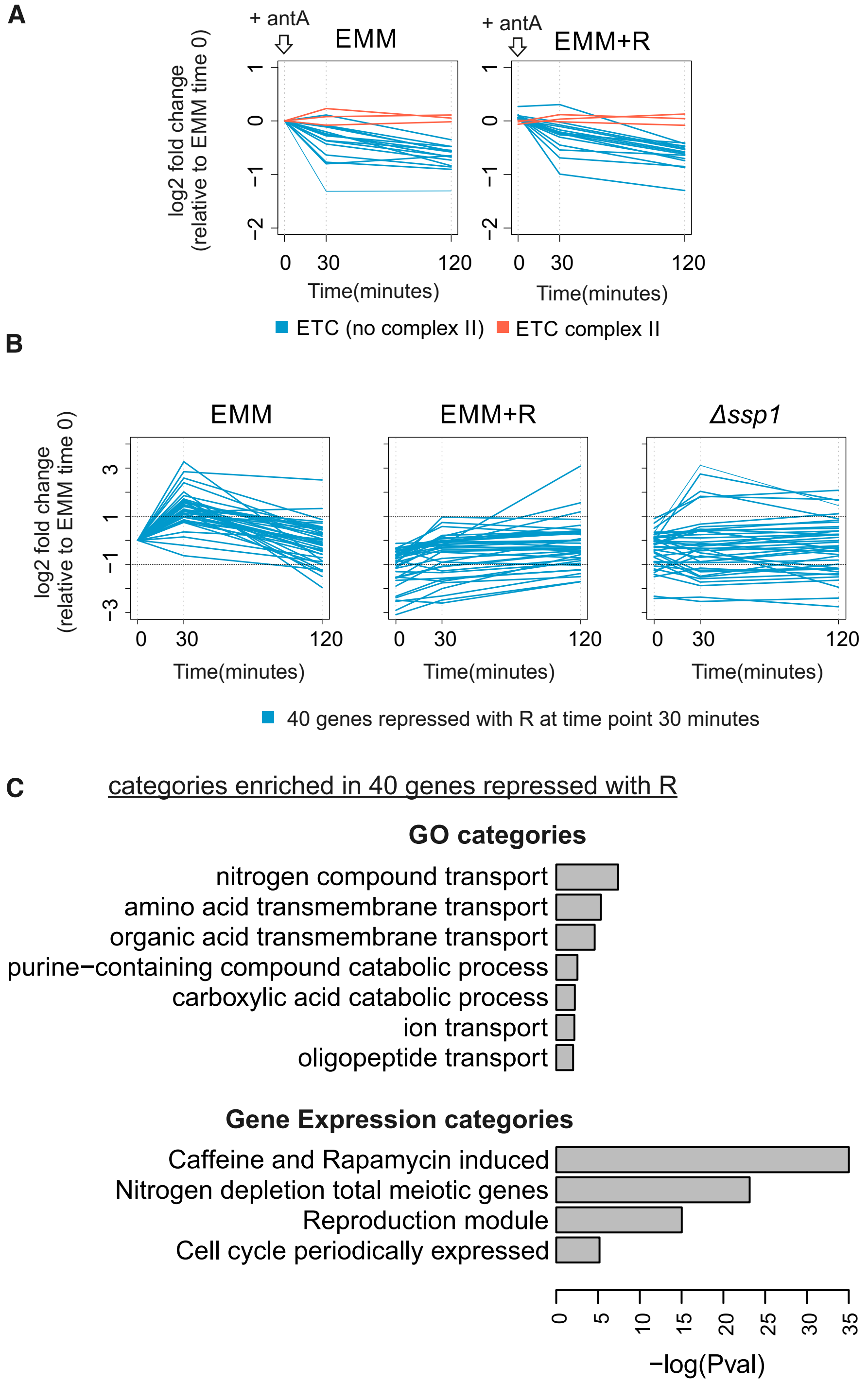

Figure EV5. Arginine does not impact repression of genes encoding electron transport chain components.

A Changes of transcript encoding electron transport chain complex subunits before and 30 and 120 min after antimycin A treatment in minimal media (EMM) and minimal media supplemented with arginine (EMM + R). Subunits of complex II are indicated in red, and other subunits in blue. Values are normalised to EMM time zero.

B Profiles of expression of 40 genes repressed in media with arginine at 30 min after antimycin A treatment. Changes of transcript abundances before and 30 and 120 min after antimycin A treatment are shown for the wild-type cells grown in minimal media with or without arginine and for *ssp1* deletion cells grown in minimal media. Values are normalised to EMM time zero.

C Selected gene ontology (GO) and gene expression categories significantly over- or under-enriched in genes repressed in media with arginine at 30 min after antimycin A treatment. Analysis performed using online tool AnGeLi (Bitton *et al*, 2015).

© 2020 The Authors
